# Supplementary material for: In vitro biomechanical evaluation of internal fixation techniques on the canine lumbosacral junction
Source: PeerJ. 2015 Aug 20;3:e1094. doi: 10.7717/peerj.1094 (PMC4548529; doi:10.7717/peerj.1094)
Supplement: Supplemental Information 1 — LS stabilization data set [file peerj-03-1094-s001.docx]

Exceptional large data sets, summaries are provided.

**Summary of Strength Failure for the Three Stabilization Techniques.**

| **Specimen** | **Group** | **Load Cycle at Failure (Nm)** | **Angle at Failure (°)** | **Failure Load (Nm)** |
| --- | --- | --- | --- | --- |
| **1** | **A** | 175 | 10.072 | 167.08 |
| **2** | **A** | 150 | 10.927 | 141.39 |
| **3** | **A** | 125 | -10.100 | -117.00 |
| **4** | **A** | 50 | -10.204 | -49.702 |
| **5** | **B** | > 350 | -- | -- |
| **6** | **B** | > 350 | -- | -- |
| **7** | **B** | 250 | -10.030 | -248.380 |
| **8** | **B** | > 350 | -- | -- |
| **9** | **C** | 150 | 10.250 | 137.468 |
| **10** | **C** | 125 | -10.327 | -125.720 |
| **11** | **C** | 125 | 10.218 | 123.15 |
| **12** | **C** | 150 | -10.436 | -146.594 |

Group A - FACET

Group B - P/PMMA

Group C – SOP

**Summary of ROM for Stabilization Techniques**

| **Biomechanical Parameter** | **Group A (FACET)** | | **Group B (P/PMMA)** | | **Group C (SOP)** | |
| --- | --- | --- | --- | --- | --- | --- |
|  | Mean | SD | Mean | SD | Mean | SD |
| **ROM-e (°)** | -3.978 | 2.870 | -0.979 | 1.872 | -1.961 | 1.520 |
| **ROM-f (°)** | -0.798 | 2.150 | 0.945 | 2.007 | 0.602 | 1.823 |
| **ROM-t (°)** | 3.180 | 1.138 | 1.924 | 0.961 | 2.562 | 0.550 |
